# Comparative Genomics of *Escherichia coli* Serotype O55:H7 Using Complete Closed Genomes

**DOI:** 10.3390/microorganisms10081545

**Published:** 2022-07-30

**Authors:** Margaret D. Weinroth, James L. Bono

**Affiliations:** U.S. Meat Animal Research Center, U.S. Department of Agriculture, Agricultural Research Service, Clay Center, NE 68933, USA; maggie.weinroth@usda.gov

**Keywords:** keyword, NGS, whole genome sequencing, genomics, microbial evolution, *Escherichia coli*, O55:H7

## Abstract

*Escherichia coli* O55:H7 is a human foodborne pathogen and is recognized as the progenitor strain of *E. coli* O157:H7. While this strain is important from a food safety and genomic evolution standpoint, much of the genomic diversity of *E. coli* O55:H7 has been demonstrated using draft genomes. Here, we combine the four publicly available *E. coli* O55:H7 closed genomes with six newly sequenced closed genomes to provide context to this strain’s genomic diversity. We found significant diversity within the 10 *E. coli* O55:H7 strains that belonged to three different sequence types. The prophage content was about 10% of the genome, with three prophages common to all strains and seven unique to one strain. Overall, there were 492 insertion sequences identified within the six new sequence strains, with each strain on average containing 75 insertions (range 55 to 114). A total of 31 plasmids were identified between all isolates (range 1 to 6), with one plasmid (pO55) having an identical phylogenetic tree as the chromosome. The release and comparison of these closed genomes provides new insight into *E. coli* O55:H7 diversity and its ability to cause disease in humans.

## 1. Introduction

*Escherichia coli* O55:H7 is a human foodborne pathogen with an unknown reservoir, although humans are thought to be the primary host [1]. O55:H7 is usually thought of as an enteropathogenic *Escherichia coli* (EPEC), but strains can also carry a Shiga toxin gene that classifies the strain as a Shiga toxin-containing *Escherichia coli* (STEC). Foodborne outbreaks from O55:H7 can have a wide range of clinical outcomes. An EPEC O55:NM outbreak in Japan resulted in cases of diarrhea [2], while a STEC O55:H7 outbreak in England had a more severe outcome, with some patients developing hemolytic uremic syndrome [3]. From an evolutionary standpoint, *E. coli* O55:H7 has been proposed as the progenitor of Shiga toxin-containing *E. coli* O157:H7 (STEC O157:H7) [4,5,6,7,8,9,10,11]. Briefly, one model of evolution [5] describes an *E. coli* O55:H7 with a pathogenicity island referred to as the Locus of Enterocyte Effacement (LEE) acquiring *stx*2 followed by the O157 O-antigen biosynthesis cluster mediating its antigen switch from *E. coli* O55:H7 to STEC O157:H7. From there, the evolutionary isolate lost its ability to ferment sorbitol and gained *stx*1, while also losing the ability to express beta-glucuronidase activity, resulting in the typical STEC O157:H7. More recently, the model was changed to show that O55:H7 strains are placed into two groups that descend from the same common ancestor as STEC O157 strains, but are not part of the stepwise evolution [6].

Whole genome sequencing of bacterial genomes has become the gold standard in both research and foodborne outbreak settings [12,13,14,15]. The ability to compare genomes at nucleotide resolution allows for the evaluation of single nucleotide polymorphisms (SNP) within genes of interest. Complex genomes with inversions and repeated regions such as mobile elements and prophage [16,17,18] cause problems in genome assemblies similarly to STEC O157:H7 based on short-read sequencing technologies. However, this problem can be addressed by long-read sequencing technologies. Integrating long-read sequencing as scaffolding with short-reads to polish the assembly and to find smaller plasmids produces a higher quality genome than either technology alone [18].

Here, we use this hybrid approach of sequencing to generate complete closed genomes from six *E. coli* O55:H7 and compare them to all other publicly available, complete closed *E. coli* O55:H7 genomes. Previously, only two O55:H7 genomes were closed and used to explore the genetic diversity of O55:H7 strains [6,9]. The six newly sequenced O55:H7 strains in this study were part of a study using multilocus enzyme electrophoresis to determine the genetic relationship between *E. coli* strains causing enteric disease [19] and were also used to understand the stepwise evolution of STEC O157:H7 [4,5,6,8]. These genomes provide a higher resolution of O55:H7 genomic diversity, including genome architecture, than previously described.

## 2. Materials and Methods

Isolate selection. A total of 10 *E. coli* O55:H7 strains were used for this study. Six were previously used to describe the phylogeny of O55:H7 and evolution *E. coli* O157:H7 strains [4,18]. The complete closed genomes from four O55:H7 strains were publicly available, with two genomes published [5,8] and two not published (Supplemental Table S1).

Wet Lab work and sequencing. All previously non-publicly available genomes that underwent sequencing were subjected to both long-read (Pacific Biosciences, Menlo Park, CA, USA) and short-read (Illumina, Inc., San Diego, CA, USA) sequencing. DNA extraction using a Genomic tip 100/g (Qiagen, Inc., Valencia, CA, USA) and size selected PacBio libraries were generated using the SMRTbell Template Prep Kit 1.0, as previously described [20]. The library was bound with polymerase P6, followed by sequencing on a Pacific Biosciences (Pacific Biosciences) RS II sequencing platform with chemistry C4 and the 360-min data collection protocol.

For Illumina library preparation, DNA from the same extraction as the long-read libraries was sheared using a microTube AFA Fiber Pre-Slit Snap-Cap 6 × 16 mm (Corvaris, Woburn, MA, USA). Libraries were created using the TruSeq DNA CPR-Free HT Library Preparation kit (Illumina, Inc., San Diego, CA, USA) and quantitated using the KAPA Library Quantification Kit (F. Hoffmann-La Roche Ltd., Basel, Switzerland) prior to pooling. The pooled libraries were run on an Illumina MiSeq with the MiSeq Reagent Kit v3 (600 cycles), resulting in 300 bp paired end read lengths.

Bioinformatics. After sequencing, raw long reads were assembled using HGAP3 [21] in SMRT analysis Version 8.0, and the resulting contigs were imported into Geneious (2020.1.2; Biomatters, Ltd., Auckland, New Zealand). If present, overlapping sequences on the ends of the contigs were removed from the 5′ and 3′ ends to generate circularized chromosomes and plasmids. The closed chromosomes were reoriented to start with the putative origin of replication using Ori-Finder 2 [22]. The closed chromosomes and plasmids were polished using the Illumina libraries via Pilon [23]. Finally, Illumina reads were mapped to the chromosome and known plasmids using Geneious, and unused reads were de novo assembled (also in Geneious) for small plasmid identification. All genomes and plasmids were annotated with the NCBI Prokaryotic Genome Annotation Pipeline [24]. Parsnp (1.2) [25] was used to generate the core chromosome phylogeny. The SNP alignment file was extracted from the Parsnp output .ggr file using HarvestTools [25]. We obtained branch supports with the ultrafast bootstrap [26] implemented in the IQ-TREE software [27] with ascertainment bias correction. The pangenome of all chromosome was visualized using Gview Server [28]. Multiple alignments were visualized using Mauve (1.1.3) [29]. EasyFig (2.2) was used to visualize plasmid similarities [30]. Sequence type, virulence genes, and plasmid types were identified using MLST 2.0, VirulenceFinder 2.0, and PlasmidFinder 2.1, respectively (http://www.genomicepidemiology.org last accessed on 17 June 2022). Insertion sequences were identified through NCBI annotation and prophages were identified with PHASTER [31]. Both were visualized with the heatmap3 package (version 1.1.9) [32] in R (version 4.0.2). Chromosome and pO55 phylogenic trees were compared using Phylo (https://phylo.io accessed on 12 May 2022) [33]. Graphical feature format (GFF) files were produced in Prokka (v1.14.6) [34] and used with Roary (3.13.0) [35] to generate set specific unique genes.

## 3. Results

### 3.1. Genomic Overview 

**Figure 1:**
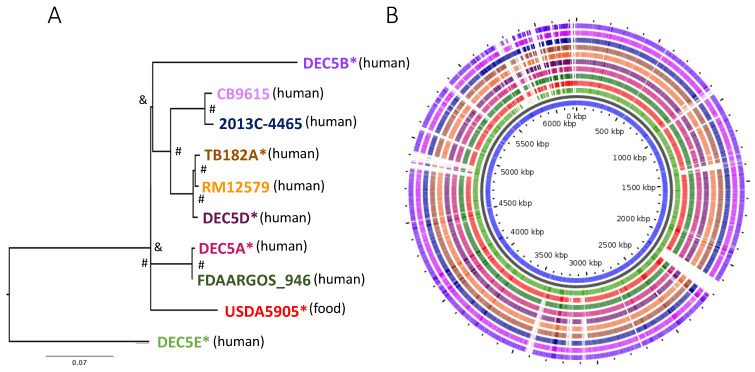
(**A**) Phylogenetic tree of the core regions from the chromosomes of *E. coli* O55:H7 strains, with newly sequenced chromosomes denoted with an asterisk (*). The tree was constructed using Parsnp with 6860 core chromosome-derived SNPs. Bootstrapping values were assigned using UFboot and SH-aLRT, with values above 95% represented by a hashtag (#) and values below represented by an ampersand (&). The source of the isolate is in parenthesis next to the strain name. (**B**) The pangenome of the chromosomes from *E. coli* O55:H7 strains using GView. The colored rings match the color of the strains at the end of the branches of the phylogenetic tree in (**A**) with blank areas representing a section of chromosome not found within a strain. The inner blue circle is the complete pangenome of the chromosomes. Strain DEC5E was used as the seed genome to build the pangenome.

Ten *E. coli* O55:H7 strains were included in the analysis: six that were subjected to long-read sequencing for the first time and four that had been previously subjected to long-read sequencing and were publicly available. Individual strain information is provided in Supplemental Table S1. The chromosomes ranged in size from 5196 kb to 5571 kb (average 5340 kb), with strains having an average of 3.1 plasmids (range 1 to 6; size average 38.8 kb, range 3.3 to 99.7 kb). The average number of protein-coding sequences (CDS) within strains ranged from 4912 to 5452 (average 5120). A phylogenomic tree of the chromosomes with 8680 core chromosomal-derived SNPs showed several groupings of O55:H7 with diverse branches (Figure 1A) using Parsnp [24]. Bootstrapping using UFboot and SH-aLRT in IQ-TREE supported the internal branches, but the branches between DEC5B and USDA5905 and USDA5905 and DEC5E were not supported.

**Figure 2:**
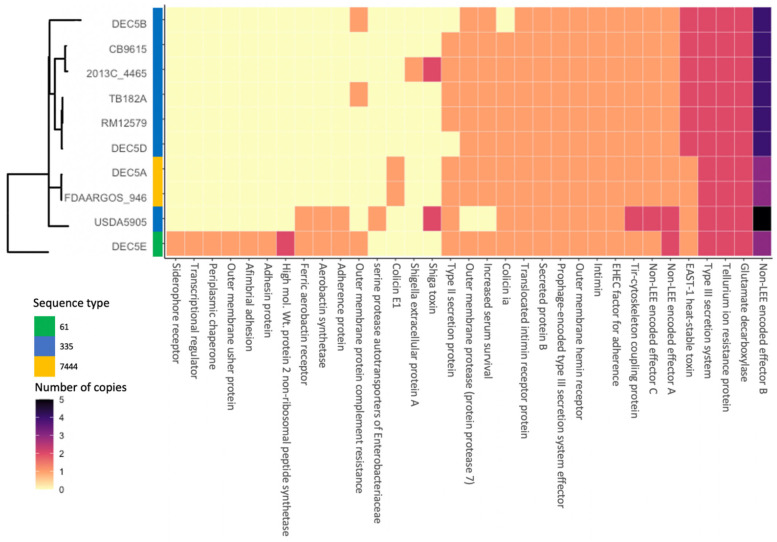
Comparison of the phylogeny of O55:H7 strain with sequence type and virulence gene content. The phylogenomic tree, strain names, and sequence type are on the *y* axis of the heatmap while the virulence factors are on the *x* axis. The number of copies of a virulence factor is represented by a different color. The phylogenomic tree is the same as Figure 1.

Figure 1B illustrates the diversity of genomic content within the serotype. Within all O55 isolates, the core genome was made up of 4273 genes. There were 193 genes only missing from one of the chromosomes and 1151 genes unique to only one isolate, with many of the unique genes belonging to strain DEC5E (inner most green ring). Multi locus sequence typing (MLST) examines the allelic profile of seven housekeeping genes to determine the relatedness of strains to each other [36]. Three different sequence types (ST) representing two different clonal complexes were identified from the 10 strains (Figure 2). DEC5E belonged to ST-61 while DEC5A and FDAARGOS-946 belonged to ST 7444. The remaining strains belong to ST 355, and all O55:H7 strains fall within a large group called ST11 clonal complex (CC ST11) (https://pubmlst.org/bigsdb?db=pubmlst_ecoli_achtman_seqdef&set_id=4&page=downloadProfiles&scheme_id=4 accessed 20 April 2022). CC ST11 also contains STEC O157:H7 strains, as would be expected, as it is the descendant of O55:H7 [37]. The main allelic difference between O55:H7 and STEC O157:H7 strains was in the *adk* gene, where O55:H7 had allele 29 while STEC O157:H7 had allele 12.

Based on the tree, DEC5E was proposed to be the earliest ancestor of the O55 isolates, with DEC5B being the most recent, and agrees with the phylogenetic analysis described by Kyle et al., 2012 [6]. When overall chromosome architecture was considered, there was a high degree of synteny (except for DEC5E which showed an inversion near the replication terminus) (Supplemental Figure S1). These large-chromosomal rearrangements (LCRs) are seen in STEC O157:H7 strains and are usually flanked by prophages that share a homologous region. RNA transcriptional profiling and phenotyping of specific structural variants indicated that important virulence phenotypes such as Shiga-toxin production, type-3 secretion, and motility can be affected by them [38]. LCRs have been seen in the chromosomes of other bacteria, including *Campylobacter*, *Staphylococcus,* and *Salmonella* [39,40,41]. In *Campylobacter*, the orientation of LCRs can provide resistance to phage infection [39]. Further research will be required to identify if the LCRs in O55:H7 strain DEC5E are related to any of its phenotypes.

### 3.2. Virulence Genes

Thirty-three virulence genes (VGs) were identified in the 10 O55:H7 strains by VirulenceFinder (Figure 2). While eighteen VGs were found in more than 80% of the strains, nine VGs were only found in one strain, five VGs in two strains and three VGs in one strain (Figure 2). DEC5E contained the most VGs with thirty, and with seven of them being unique to this strain. The least number of VGs was eighteen, and was shared by five strains: DEC5B, CB9615, TB182A, RM12579, and DEC5D. DEC5E and USDA5905 shared three VGs that encode for the ferric aerobactin receptor, aerobactin synthetase, and an adherence protein. Aerobactin is used to acquire iron and enhance biofilm formation, oxidative stress resistance, and virulence in *Yersinia pseudotuberculosis* [42]. DEC5E also carried an additional siderophore receptor that binds iron, two more adhesion proteins, and a complement resistance protein. The combination of shared and strain-specific virulence genes indicated that this strain might be more infective than the other O55:H7 strains. This hypothesis would need to be tested to determine its validity, which is beyond the scope of this paper. In addition to the aerobactin genes and adherence genes shared with DEC5E, USDA5905 contained the *stx2_d_* Shiga toxin variant. Shiga toxin is a virulence factor that was originally identified in *Shigella dysenteriae* but has been found in other bacteria, including several *E. coli* serogroups [43]. Stx2d is one of the most potent Shiga toxin variants along with Stx2a. DEC5A and FDAARGOS-946 belong to the same phylogenomic clade and share one VG found only in this group. Colicin E1, a protein that can puncture the bacterial cell wall, causing cell death, was found on a 6-kb plasmid unique to these strains. As previously described, USDA5905 contained the Stx2d variant, while strain 2013C-4465 had the Stx1avariant, the least potent of the two. Colicin 1A was found in 9 of the 10 strains. DEC5B lacked this gene because it did not carry the pO55 plasmid. All 10 strains contained proteins encoded by VGs that are shared with STEC O157:H7, a progenitor of O55:H7. These include the translocated intimin receptor, intimin, EHEC factor for adherence, non-LEE encoded effectors, tellurium resistance, and glutamate decarboxylase. All strains possessed multiple copies of the *nleB* gene, which encodes for the non-LEE encoded effector B protein, in their genomes. USDA5905 has five copies of this gene, while DEC5A, FDAARGOS-946, and DEC5E each had three. The remaining strains had four copies. These results differ from those of the STEC O157:H7 strain Sakai, which only contains two *nleB* genes. The *nleB*-encoded protein is thought to inhibit proinflammatory signaling and necroptosis [44] and has been identified as one of three proteins essential for effective colonization [45,46,47]. Of the 33 VGs identified, 14 proteins encoded by VGs were identified in all strains from human cases, while 19 were found in single or multiple strains, but not in all strains. The VGs not identified in all the strains could increase the infectivity or severity of the disease depending upon the combination. Another factor to consider when trying to determine virulence is the immune status of the host. Humans with a compromised immune system might be more susceptible to a pathogen with few virulence factors.

### 3.3. Prophages

**Figure 3:**
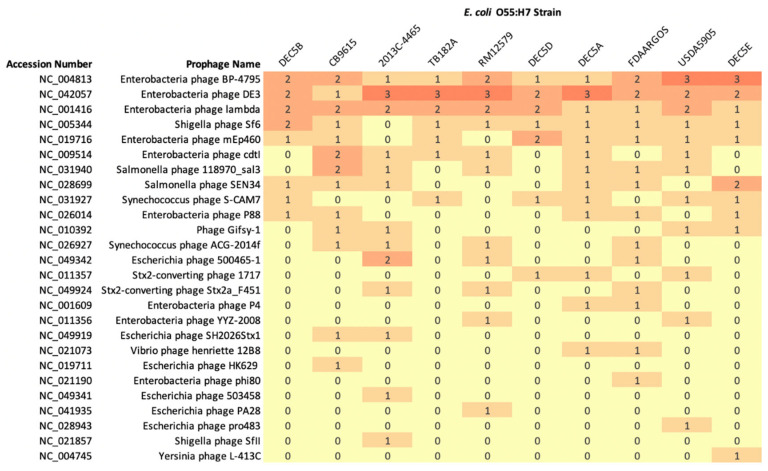
Heatmap of the number of prophages presents in *E. coli* O55:H7 chromosomes with the number of phages also included in the cell. The prophage accession number and name are on the *y* axis, while the strain names are on the *x* axis. The number of copies of a prophage is represented by a different color.

PHASTER can predict if a prophage is complete, incomplete, or questionable based on prophage similarity using BLAST+ [31]. However, prophages predicted by PHASTER with the same name may have different genetic structures. The comparison of prophage content between O55:H7 strains reported the predicted classification, and is not based on prophage structure similarity. Chromosomes were determined to be comprised of 10.0% prophage content (range 7.6 to 12.4%) and encoded between 10 and 17 phages (average 14.2) (Figure 3).

Three prophages were present in all genomes (sometimes in multiple copies): Enterobacteria phage BP-4795 (NC_004813), Enterobacteria phage DE3 (NC_042057), and Enterobacteria phage lambda_(NC_001416). Enterobacteria phage BP-4795 has been characterized to contain two IS*629* elements and a type III secretion system effector, NleA_4795_ [48]. There were seven phages unique to one isolate, while other phages were only found in strains grouped by phylogeny. For example, *Escherichia* phage SH2026Stx1 was found in strain CB9615 and 2013C-4465, while phages Enterobacteria phage p4 and *Vibrio* phage Henriette 12B8 were found in strain DEC5A and FDAARGOS-946 (Figure 3).

Two strains, USDA5905 and 2013C-4465, had prophages that encode the Shiga toxin genes, *stx2_d_* and *stx1_a_*, respectively. *stx2_d_*-encoding prophages were extracted from nine closed genomes deposited in NCBI and were compared with the *stx2_d_*-encoding prophage from USDA5905 (Supplemental Figure S2A). The *stx2_d_*-encoding prophages could be placed into one of five groups. Prophages shared regions of homology within each serogroup but shared little homology with different serogroups. Six of the prophages, from strains USDA5905, NCCP15955, STEC 313, STEC 316, STEC 367, and STEC 1025, were integrated into *yecE*, a gene with an unknown function [49,50]. Integration of a *stx2_d_*-encoding prophage at *yecE* is not unique and has been observed in STEC serotype O26:H11 [51]. The *yecE* gene is one of the six sites that *stx*-encoding prophages are known to integrate. Another integration site is *yehV*, a gene encoding a transcriptional regulator [48,52]. The *stx2_d_* prophage in RM10410, an *E. coli* serotype O111:H4, is integrated into this site. The integration site of *stx2_d_* prophages in strains M7424, M00057, and M11957 was at a site not previously recognized as a site of integration for *stx*-encoding prophages. The prophage was integrated between the *zinT* and *mtfA* genes. This integration site was occupied in other O55:H7 and STEC O157 strains with a non-*stx*-encoding prophage (Supplemental Figure S3). In STEC O157:H7 strain NE1169-1, a tellurite resistance protein was in place of the *stx2_d_* gene, while O55:H7 strain USDA5905 had a hypothetical protein and DUF1327 domain-containing protein in this site. A phylogenetic tree of the *stx2_d_* genes identified four different variants (Supplemental Figure S2B). The *stx2_d_* gene from USDA5905 was one identified variant, while the *stx2_d_* genes RM10410 and NCCP 15955 formed a second variant. The third *stx2_d_* variant was found in strains M7424, M00057, and M11957, with the fourth *stx2_d_* variant in strains STEC313, STEC316, STEC367, and STEC1025. The grouping of the *stx2_d_* phylogenetic tree reflects the same grouping in the *stx2_d_* prophage alignment, demonstrating the specificity of the *stx2_d_* gene to the prophage that carries it.

The *stx1_a_*-encoding prophage was extracted from six closed STEC O157:H7 genomes deposited in NCBI and was compared to the *stx1_a_*-encoding prophage from strain 2013C-4465 (Supplemental Figure S2C). The 2013C-4465 *stx1_a_*-encoding prophage was integrated into *argW,* which encodes the transfer RNA, tRNA-Arg [53,54], while the six *stx1_a_*-encoding prophages from STEC O157:H7 were integrated into the *yehV* site. When comparing the prophage structure, the STEC O157:H7 prophages were placed into one of two groups while the 2013C-4465 prophage structure was grouped by itself. This demonstrated that prophage-encoding *stx1_a_* genes are significantly different structurally but contain *stx1_a_* genes with identical sequence. This agrees with the presence of the *stx1_a_*-encoding prophage in STEC O157:H7 strain in UK [55]. Prophage-encoding *stx1_a_* did not share a high level of similarity across lineages, geographical regions, or time, but shared similarity at the gene level. The two STEC O157:H7 *stx1_a_*-encoding prophage groups were associated with a polymorphism in the *tir* gene that associates with a strain’s ability to cause disease in humans [53]. This same grouping can be seen in the phylogenetic tree of the *stx1_a_* genes (Supplemental Figure S2D). The *stx1_a_* gene from 2013C-4465 was identical to the *stx1_a_* genes from STEC O157:H7 *tir* 255T allele that associates with human disease. In STEC O157:H7, 98% of clinical isolates from humans have the *tir* 255T allele, while 2% have the *tir* 255A allele [53]. The strains carrying the *tir* 255A allele are proposed to have diverged from strains carrying *tir* 255T early in the evolution of STEC O157:H7. This suggests a possible lineage specificity of *stx1_a_*-encoding prophages in O55:H7 and STEC O157:H7 strains depending upon the *tir* 255 allele variant.

### 3.4. Insertion Sequences

**Figure 4:**
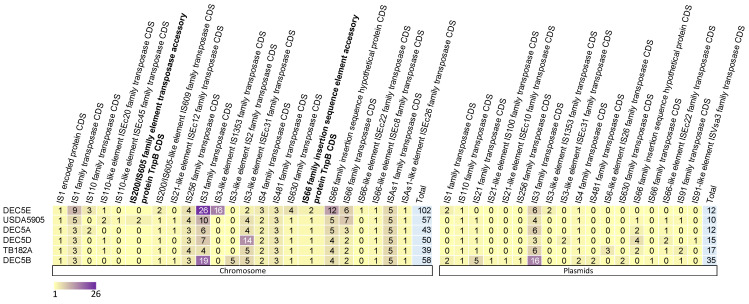
Heat map of insertion sequence (IS) elements in the chromosome and plasmids of six *E. coli* O55:H7. Plasmids are not broken down by individual plasmids; instead, all plasmids present in an isolate are aggregated into one. The strain names are on the *y* axis with the IS elements on the *x* axis. The number of copies of an IS element is represented by a different color.

Insertion sequence elements (IS elements) were specifically evaluated between a subset of isolates (those with common annotation in Genebank) shown to play a role in *E. coli* O157:H7 diversification in the population, specifically IS629 [56]. Across the six isolates studied, 450 IS elements were identified: 349 within chromosomes and 101 within plasmids (Figure 4).

On average, within the chromosome of O55:H7, there were 55 (ranging from 39 to 102) IS elements, while the plasmids had an average of 16 IS elements (ranging from 10 to 35). Eight IS elements were unique to one isolate, and eight were found in at least one copy within all isolates (six on the chromosome and one on the plasmids). The IS3 family transposase was found in all isolates on both the chromosome and plasmids in the highest copy number. One other IS element, IS4 family transposase, was found within at least one plasmid in all strains.

IS elements are drivers of evolution and diversity in bacteria. In STEC O157:H7, there are multiple reports of the Shiga toxin gene being inactivated by IS elements. In the strain DEC5B, there are three genes encoding for *citC*, fimbrial biogenesis outer membrane usher protein, and autotransporter outer membrane beta-barrel domain-containing protein that have IS elements inserted into them, presumably inactivating these genes. Two of these genes have an inserted IS3 family transposase, while one has an IS3-like element ISEc31 family transposase. *citC* is part of an operon that is responsible for citrate fermentation and would presumably not function in DEC5B. Recently, a novel overlapping, open reading frame was identified internal to the *citC* gene. The novel protein, Nog1, is thought to provide a growth advantage in the presence of MgCl_2_ and is transcribed about 14-fold higher in cow dung compared to Luria broth [57]. Since these genes are intact in STEC O157, the proposed progeny of DEC5B, the IS elements were either excised or STEC O157 descended from another closely related strain missing the inserted IS elements.

### 3.5. Plasmids

Plasmid carriage was diverse, with 31 plasmids found between all isolates that grouped into six common backbones. Five of the strains carried an antibiotic resistance cassette, including two strains that had multiple cassettes. All plasmids were placed into one of six groups except plasmids pUSDA5905-1 and p12579-1 that appeared to be similar bacteriophages. All plasmids except pUSDA5905-1 and pUSDA5905-5 were circularized by trimming overlapping 5′ and 3′ end to make a closed circle.

Plasmid pDEC5B-3 was highly related to plasmids pUSDA5905-3, P12579-4, TB182A-3, and pDEC5D-2 (Supplemental Figure S4A). DEC5B-3 was 3.3 kb longer than the other related plasmids and had an additional *sul2* gene, while DEC5D-2 had an IS91-like element in place of the *aph*(3′)-Ib and *aph*(6)-Id antibiotic resistance genes. Interestingly, pTB182A-3 and pTB182A-5 share the same antibiotic resistance cassette, *sul2*, *aph*(3″)-Ib, and *aph*(6)-Id, indicating again the potential for this strain to have increased resistance to these antibiotics. These plasmids contain a plasmid replication initiation gene whose protein product recruits and positions an active helicase at the plasmid replication origin [58].

Plasmids pTB182A-2, pDEC5B-2, pDEC5A-3, and pFDAARGOS-3 are between 5.4 kb and 6.8 kb in size and share just over 3 kb of homology to each other (Supplemental Figure S4B). Plasmids pDEC5B-2 and pTB182A-2 are the same size and only differ by nine bases. The genes found in the related regions were *mobC* and *mbeA,* whose encoded proteins are multifunctional and promote conjugal plasmid mobilization [59,60]. These plasmids are missing the *mobB* gene, which is part of the mobilization operon. If present, these strains could use the conjugation system (*tra* operon) on an IncF plasmid in the cell to be horizontally transferred to other strains. Plasmids pDEC5B-2 and pTB182A-2 possess a nickel transport and two hypothetical genes in the non-conserved region, while pDEC5A-3 and pFDAARGOS-3 have a colicin 10 operon.

pDEC5A-3, pFDAARGOS-2, and pUSDA5905-5 are small plasmids with a roughly 1.7-kb homologous region that contains the gene *nikA*. The NikA protein from the plasmid R64 combines with NikB to form a relaxation complex at the *oriT* region of the plasmid and prepares the plasmid to be replicated [61]. The relaxation complex and replication may not function in these plasmids because there is no annotated *nikB* gene or other genes involved in plasmid replication. (Supplemental Figure S4C). pDEC5A-3 and pFDAARGOS-2 are identical and contain an ATP-binding protein in the non-conserved region, while plasmid pUSDA5905-5 has an RNA-directed DNA polymerase. A group of plasmids was only found in strains USDA5905 and RM12579.

Plasmids p12579-3, p12579-5, and pUSDA5905-4 have homology with a conserved region of approximately 3.0 kb that contains the complete plasmid mobilization operon *mobABC*. The proteins encoded by this operon are required for formation of the relaxasome for mobilizing the small plasmid (Supplemental Figure S4D). Like the previously described plasmids, these plasmids are in strains that contain an IncF plasmid that contains the *tra* operon for transferring mobile elements between strains and is compatible with mobilizing plasmids with *mobABC* [62]. This indicates the potential of these small plasmids to be horizontally transferred to other strains. The IncF plasmid p12579-3 has three hypothetical genes, a transposase, and a beta-lactamase TEM antibiotic resistance gene in the non-conserved region, while p12579-5 only has two hypothetical genes. pUSDA5905-6 only has a hypothetical gene in the non-conserved region, while pUSDA5905-4 has a site-specific methyl transferase and restriction endonuclease genes.

pDEC5B-5 was most similar to plasmids pDEC5E-2 and TB182A-5 (Supplemental Figure S4E). These plasmids range in size from 70 kb to 99 kb, with the defining feature being the *tra* conjugation transfer systems. While this region appears to be conserved, the non-conserved regions are quite different. Plasmid pDEC5B-5 has 16 IS elements and a translesion error-prone DNA polymerase (*umuC* and *umuD*), while pTB182A-5 contains antibiotic resistance genes TEM-1, *sul2*, *aph*(3″)-Ib, *aph*(6)-Id, and *dfrA8*. pDEC5E-2 contains the previously described antibiotic resistance region and a mercury resistance operon. These plasmids belong to the IncFII plasmid incompatibility group.

**Figure 5:**
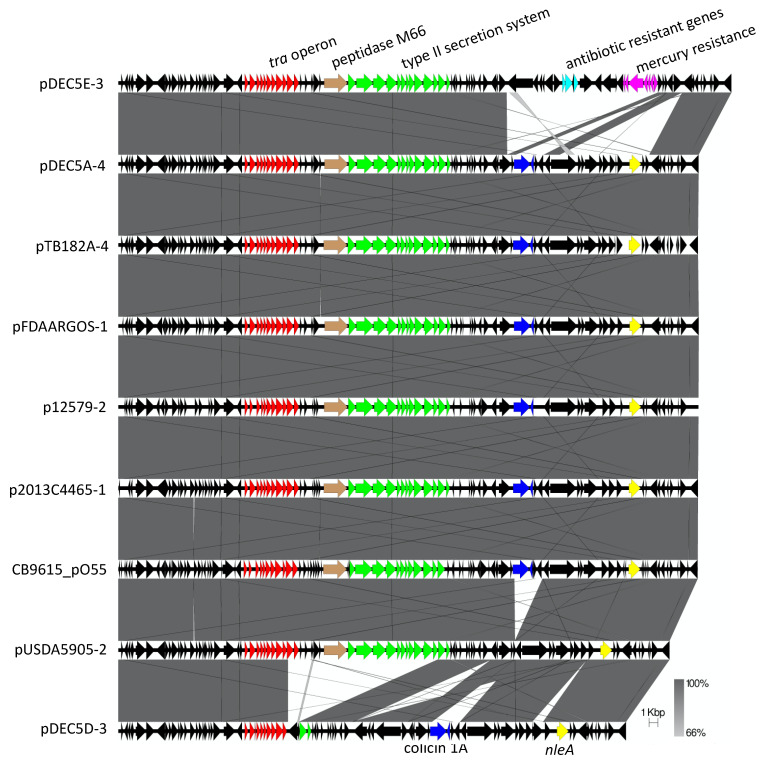
Genetic map showing the O55:H7 57-kb to 69-kb plasmid present in 9 of the 10 strains. Each colored arrow represents a different gene or group of genes labeled above or below the genetic map. The black arrows are other genes on the plasmids. The shades of gray between the genetic map represents the percent similarity of the plasmids to each other. The scale bar represents plasmid size in kilobases.

A 57-kb to 69-kb plasmid (pO55) was found in all strains except for DEC5B (Figure 5). This plasmid belongs to the IncFIB incompatibility group and shares similar regions with the pO157:H7 plasmid in STEC O157:H7 [9]. However, unlike pO157, whose plasmids are almost identical except for a few IS elements, there were four variants of pO55. The differences were due to deletions, insertions or a replacement of a region with other genes. The most common genes shared by pO55 and pO157 were for conjugation, a type II secretion system, a colicin, and a type III secretion system effector, *nleA*. Interestingly, the plasmid pDEC5D-3 was missing the type II secretion system and colicin genes, while the plasmid pDEC5E-3 also contains the antibiotic resistance genes *aadA1*, *qacE*Delta1, and *sul1*, along with an operon for mercury resistance. This 16kb resistance region was also found on the pDEC5E-2, another plasmid in DEC5E, indicating the potential of increased resistance to these antibiotics. pDEC5E-2 also contains a type A-1 chloramphenicol resistance gene. The pO55 plasmid appears to evolve at the same rate as the chromosome. When the phylogenetic trees of the chromosome and pO55 were compared, the structure of the subtrees trees matched at its best corresponding node. (Supplemental Figure S5). This result is similar to STEC O157:H7, the proposed descendant of O55:H7. Nyong et al. showed a stable evolutionary relationship between the host chromosome and pO157 plasmid [20]. Plasmids, being mobile elements, are thought to be transient in bacteria. However, there are now examples in two *E. coli* serotypes where plasmids have taken on the role of extrachromosomal elements and are stably maintained in the population.

Strain DEC5B was the lone O55:H7 isolate that did not contain a pO55 plasmid. Plasmid pDEC5B-4 belongs to the IncFII incompatibility group and was the closest plasmid in DEC5B that resembled pO55 or pO157. (Supplement Figure S6). The homology between pDEC5B-4 and pO157 includes several hypothetical genes and conjugation genes (*tra*) (Supplement Figure S6A). DEC5B-4 shows the most similarity to pDEC5B-5, another plasmid in DEC5B, pDEC5E-2, and TB182A-2. This region was about 11 kb in length, with most of the similarity with the *tra* genes (Supplement Figure S6B). Comparison of pDEC5B-4 to pO55 shows homology with the same hypothetical genes as pO157, but to a different region of the *tra* operon (Supplement Figure S6C). Interestingly, DEC5B was the strain most closely related to STEC O157:H7 strains [6] and had the same sequence type as five other O55 strains. However, it did not contain a plasmid that was similar to either pO55 or pO157. This suggests that at some time this strain lost the pO55 plasmid and acquired plasmid pDEC5B-4, or this strain belongs to a lineage of O55:H7 strains that never acquired plasmid pO55. While strain DEC5B is currently the closest known ancestor to STEC O157:H7, there is a yet to be discovered strain from which the STEC O157:H7 lineage descended.

### 3.6. Comparison of Genes in O55:H7 Complete Genomes

Using Roary, the gene content of the 10 O55:H7 strains was compared based on phylogenetic tree placement (Figure 1). Three strains, DEC5E, USDA5905, and DEC5B, were the lone occupants at branch tips and were compared as individuals. The rest of the strains could be placed into one of three groups: group 1—DEC5A and FDAARGOS_946, group 2—DEC5D, RM12579 and TB182A, and group 3—CB9615 and 2013C-4465 (Supplemental Table S4). DEC5E had the most unique genes when compared to the other strains (*n* = 503), with 73 unique to the plasmids and 430 unique to the chromosome. Group 2 had the least unique genes (*n* = 42), with all of them on the chromosome. Most of the unique genes in these comparisons were hypothetical proteins or phage-related and were classified as belonging to gene families (Supplemental Table S4).

The Roary output classified duplicated genes caused by a missense mutation that disrupted the open reading frame. Some of the mutated genes provided essential functions for the bacterium (Supplemental Table S4). For instance, in DEC5E, a base substitution in the *eutA* gene created a truncated protein. The *eutA* gene is part of the operon for ethanolamine utilization. Ethanolamine catabolism is associated with bacterial pathogenicity in *S. Typhimurium*. Transcript expression studies link the increase in *eut* expression with the activity of global regulators including CsrA and Fis [63]. The DEC5E strain was isolated from a human specimen, indicating that in this situation a complete *eutA* gene was not needed for causing human disease. DEC5E does contain seven virulence factors that were not found in the other O55:H7 strains and four that were shared with two or three other strains, so the possibility exists that these factors were able to overcome the loss of *eutA* and cause disease in this individual.

The chromosome of USDA5905 contained a missense mutation in the *rssB* gene that encodes for the response regulator RssB. RssB acts as a proteolytic recognition targeting factor for RpoS, a stationary phase sigma factor that controls many genes involved in helping cells deal with the stresses from being in a stationary growth phase. RpoS is regulated at the transcriptional and translational levels, but RssB regulates RpoS by specifically targeting the protein for degradation by ClpXP [64]. *rssB* mutants express high levels of RpoS and impaired osmotic regulation and stationary phase response [65]. In *S. typhimurium*, inactivation of *mviA*, the homolog of *rssB* in *E. coli*, caused a growth defect resulting in small colonies and attenuated virulence [66]. While no colony morphology difference was noted between USDA5905 and the other O55:H7 strain, additional studies would be needed to determine if this strain has similar phenotypes to the *S. typhimurium mviA* mutant.

Throughout group 1, there were 127 unique genes, with 8 found on plasmids and 121 on the chromosome. This group of strains possessed a truncated *uidB* gene created by a missense mutation at bp 1168 (G-T) that resulted in a protein that is 68 amino acids shorter than the other strains. UidB is a proton-dependent transporter for α- and β-glucuronides, transporting them from the environment into the cell where they are cleaved to yield glucuronate [67]. Glucuronate is then available as a source of carbon for growth. In most STEC O157:H7 cells, the UidA protein is truncated by an insertion in the *uidA* gene [68]. The result of this inactivated protein provides one of the key phenotypic diagnostic features of this serotype. The inability of UidA to cleave β-glucuronides substrates that produce a color or fluorescence, along with the inability to ferment sorbitol, is used in different methods to identify potential STEC O157:H7 isolates. Group 1 strains appear to share the same UidA phenotype as STEC O157:H7, as the truncated UidB protein would not be able to import α- and β-glucuronides into the cells allowing UidA to cleave them. The loss of the ability to use α- and β-glucuronides as a carbon source does not appear to prevent O55:H7 group 1 strains and STEC O157:H7 strains from causing disease in humans.

Groups 2 and 3 did not have any unique gene in their plasmids and had a total of 43 and 46 unique genes, respectively, on the chromosome. For Group 2 strains, sulfoquinovose isomerase, a gene in the sulfoquinovose degradation I system, had a thymidine inserted at bp 482, creating a truncated protein. The insertion of a base may be caused by a sequencing error. However, this insertion was present in all three strains of this group which were sequenced independently in three different labs. The strain sequenced from our group was verified by mapping short-read sequences to the insertion site. Sulfoquinovose (SQ) is one of the most abundant organic sulfur compounds in nature and is found in many plants. Sulfoquinovose isomerase is the first enzyme in the Embden-Meyerhof-Parnaas pathway, and converts sulfoquinovose to sulfofructose [69]. The end products of the pathway are dihydroxyacetone phosphate, which provides energy for growth, and 2,3-dihydroxypropane sulfonate, which is exported from the cell. In culture, *E. coli* strain K-12 can use SQ as a sole source of carbon and energy. It is proposed that this pathway provides a source of bacterial energy in the intestinal tracts of humans and animals where available sources of metabolites are limited [70]. The inactivation of this pathway in Group 2 strains would suggest that these strains may not grow as vigorously as strains with a complete pathway.

In Group 3, the *pgaB* had a single bp deletion at bp 778 of the gene. This region of the gene has a stretch of seven adenosines in the full-length gene but only six in the two strains from Group 3. Strains CB9615 and 2013C-4465 were sequenced by other groups, so the deletion could not be verified. However, this deletion was not seen in the other strains in the study and were independently sequenced by two groups, so we assume the deletion is real. The *pgaABCD* operon affects biofilm development by promoting abiotic surface binding and intercellular adhesion by synthesizing and exporting poly-N-acetyl glucosamine (PNAG). All genes in this operon are required for optimal biofilm formation [71]. In uropathogenic *E. coli*, the *pgaABCD* operon is required for fitness in a mouse model of bacteremia and urinary tract infection and promotes biofilm formation in uropathogenic *E. coli* [72]. The inability to make PNAG in strains CB9615 and 2013C-4465 leaves them at a disadvantage during competition with other bacteria for resources in the environment and potentially reduces their ability to cause disease in humans.

Lastly, DEC5B had 288 unique genes, with 149 unique to the plasmids and 139 unique to the chromosome. In DEC5B, the *malE* gene contained a missense mutation at bp 551 (G-A) that created a truncated MalE protein. MalE is a maltose/maltodextrin ABC transporter substrate-binding protein responsible for delivering maltose or maltodextrin to the transport complex for internalization into the cell. Maltose is then cleaved by an amylase to release two glucose molecules for glycolysis. Maltose has been shown to be important for colonization of pathogenic and commensal *E. coli* strain in the intestines of mice [73]. Without the ability to utilize maltose, DEC5B might be at a disadvantage when colonizing the intestines of humans and thus reducing their ability to cause disease.

Nine of the ten strains used in this study were isolated from humans, while USDA5905 was isolated from food (meat). Interestingly, there were truncated genes in each of the human strains that were involved in metabolism, colonization, or the ability to cause disease in humans. This indicates that the other virulence factors in the strains were able to overcome the loss of function from these inactivated genes. However, we do not know much about the immune or health status of the humans that were infected with these strains. Additional research would be needed to understand how the combination of virulence factors and other genomic diversity of these strains affect their ability to cause disease.

## 4. Conclusions

This study leveraged the complete closed genomes from 10 *E. coli* O55:H7 strains to look at the diversity of these human pathogenic strains. The strains were assigned to three clades. Differentiation of clades could be attributed to sequence type and virulence gene profile but not to the chromosome architecture, which was similar except for one strain. Prophage and insertion sequence content did not associate with clade assignment or sequence type. Two strains contained a prophage that encoded for two different Shiga toxin genes, *stx1_a_* and *stx2_d_*. The O55:H7 prophage containing *stx1_a_* had a different gene structure than those from STEC O157:H7, but the *stx1* gene was identical to a STEC O157:H7 *stx1* gene. The O55:H7 *stx2_d_*-containing prophage and the *stx2_d_* gene were not similar to *stx2_d_*-containing prophages or *stx2_d_* genes from other serotypes. The O55:H7 strains contained many plasmids that did not associate with clade or sequence type. These plasmids could be classified into eight groups with one group closely related to prophages. There were several strains that contained plasmids with multiple copies of the same antibiotic resistance cassettes and mercury resistance operons, indicating the potential of these strains to be resistant to a higher concentration of these antimicrobials. Plasmid pO55 was found in nine of the ten strains. This plasmid showed high homology across the length of the plasmid except in two strains with an insert, with antibiotic and heavy-metal resistance in one strain and a deletion of the type II secretions system in the other strain. The phylogenetic tree from the core genome of this plasmid had the same branching pattern as the chromosome, indicating that pO55 has a stable evolutionary relationship with the chromosome. Finally, missense mutations in genes related to metabolism, colonization, and virulence factors were identified that associated with clade assignment. Despite the diverse genome, these O55:H7 strains were still able to cause disease in humans.

## Data Availability

All newly sequenced fastq files and assemblies have been submitted to the National Center for Biotechnology Information (NCBI) under BioProject PRJNA528413.

## Supplementary Materials

The following supporting information can be downloaded at: https://www.mdpi.com/article/10.3390/microorganisms10081545/s1, Figure S1: Mauve alignment of all *E. coli* O55:H7 chromosomes, Figure S2: Comparison of *stx*-encoding prophages in O55:H7 to related prophages and *stx* genes, Figure S3: Integration site of *stx2_d_* prophage in O17/O44:H18 compared to the prophages integrated into the same site in O55:H7 and STEC O157:H7, Figure S4: Additional plasmid identified in O55:H7 strains, Figure S5: Comparison of pDEC5B-4 to pO157, pO55, and additional pO55 plasmids, Figure S6: Comparison of the phylogenetic trees from the chromosome and pO55 plasmids from O55:H7. Table S1: Strains used in the study with associated metadata, Table S2: The prophage content of each strain as determined by PHASTER, Table S3: The insertion element content of *E. coli* O55:H7 strains as determined by NCBI annotation, Table S4: The unique gene content of *E. coli* O55:H7 strains as determined by Roary.
