# Supplementary material for: Comparative Genomics of Escherichia coli Serotype O55:H7 Using Complete Closed Genomes
Source: Microorganisms. 2022 Jul 30;10(8):1545. doi: 10.3390/microorganisms10081545 (PMC9413875; doi:10.3390/microorganisms10081545)
Supplement: Supplementary file 1 [file microorganisms-10-01545-s001.zip › O55_Suppl_Figures_Genomes_Microorganisms.pdf]

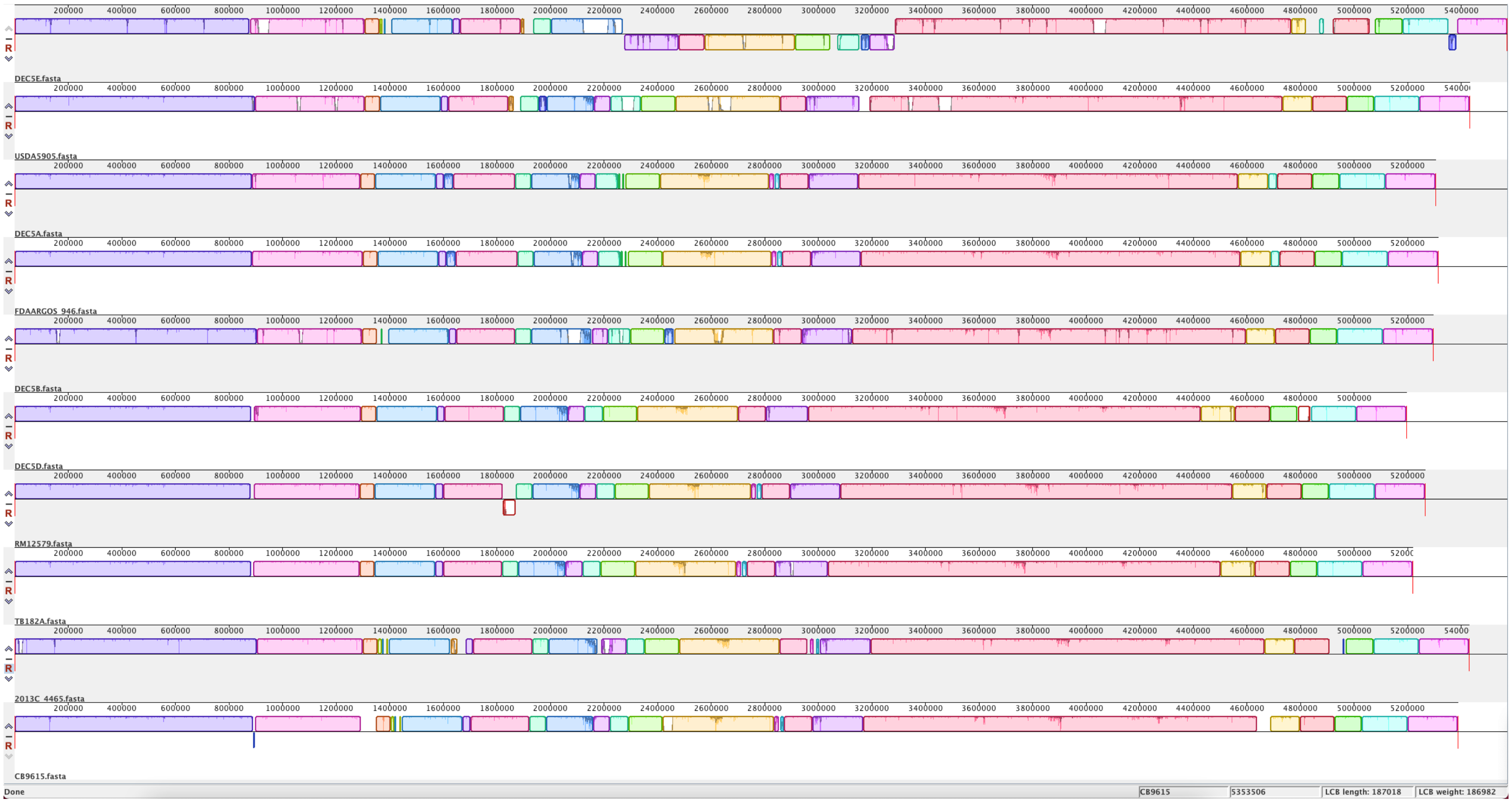

Supplemental Figure S1. Mauve alignment of all *E. coli* O55:H7 chromosomes. Changes in color within a panel indicates conserved regions between the chromosomes while lines within one color indicate nucleotide or small indel differences of one chromosome from the others. Colored pieces of the chromosomes descending below the top line indicted an inversion of that section of the chromosome in relation to others in the clade. The overall architecture of the chromosome from each isolate are highly homologous with the one notable difference of an inversion in DEC5E.

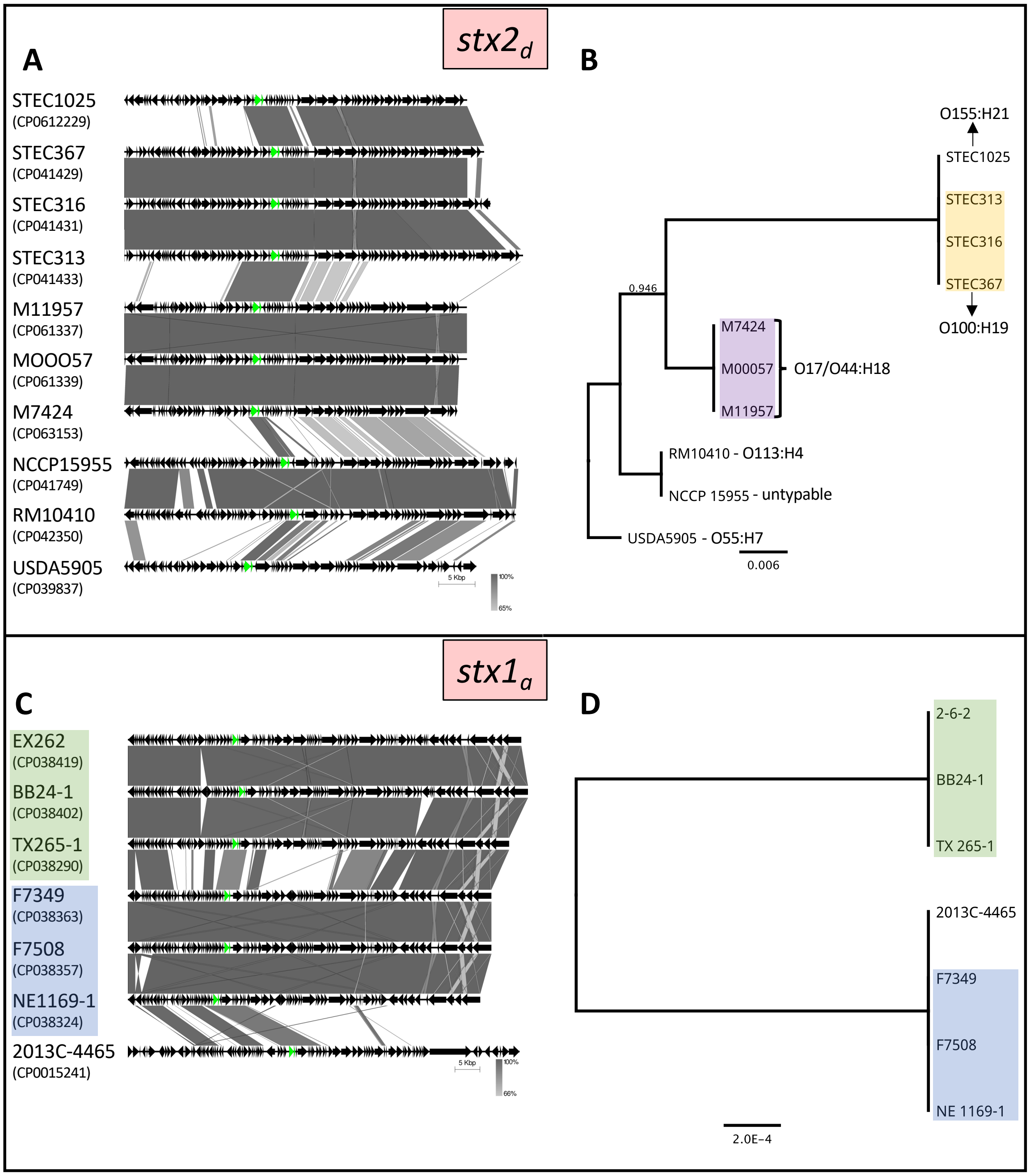

Supplemental Figure S2. Comparison of *stx* encoding prophages in O55:H7 to related prophages and *stx* genes. A) Comparison of the $stx2_d$ encoding phage in O55:H7 strain USDA5905 with other prophages containing $stx2_d$. The Genbank accession number of the strain is located below the strain name. B) phylogenetic tree of the $stx2_d$ genes from various serotypes. The O and H type are indicated next to the strain names. C) Comparison of the $stx1_a$ encoding prophage from O55 strain 2013C-4465 to $stx1_a$ encoding prophage from STEC O157:H7 strains. The Genbank accession number of the strain is located below the strain name. D) phylogenetic tree of the *stx* genes from O55:H7 and STEC O157:H7. The green arrow in the prophage alignment indicates the position of the *stx* gene. The green highlighted strain names have the *tirA* allele while those in blue have the *tirT* allele.

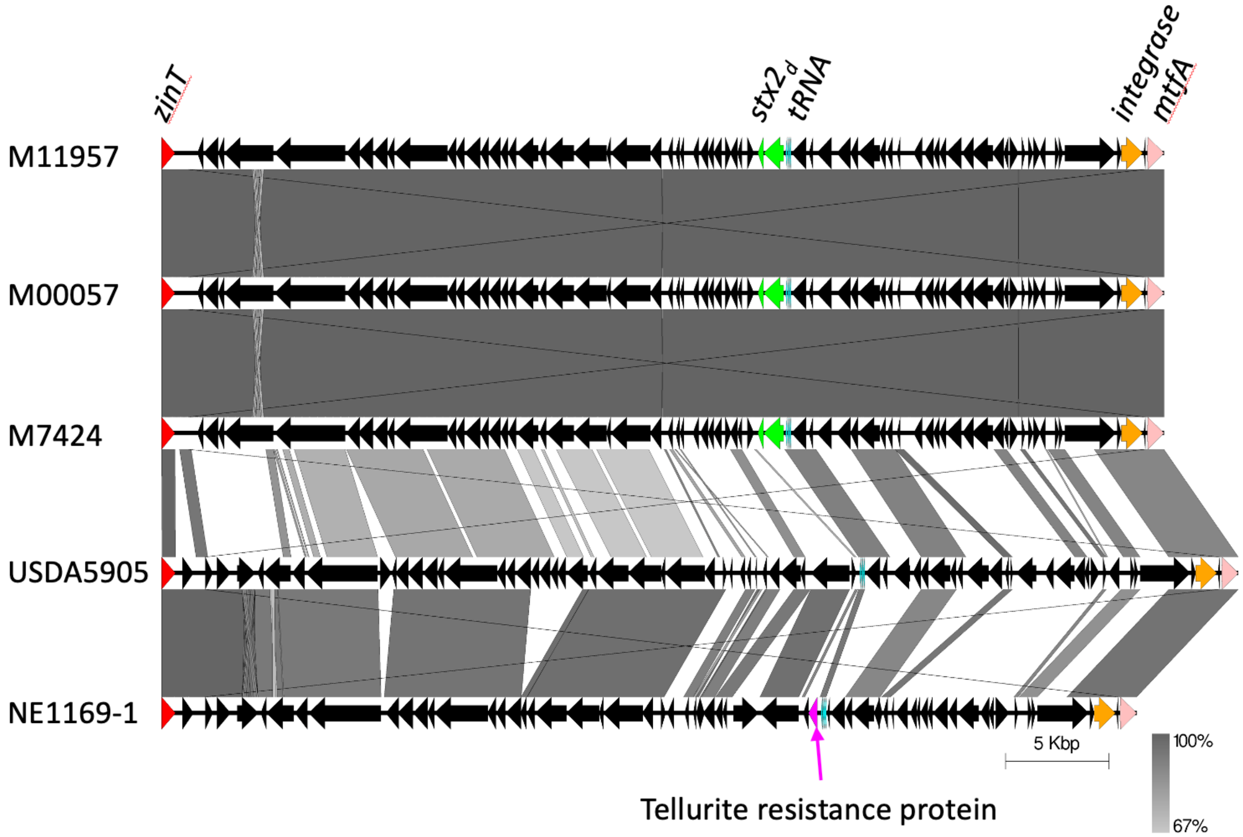

Supplemental Figure S3. Integration site of *stx2$_d$* prophage in O17/O44:H18 compared to the prophages integrated into the same site in O55:H7 and STEC O157:H7. Strain names are on the left with strains M7424, M00057, and M11957 belonging to serotype O17/O44:H18. Strain USDA5905 is serotype O55:H7 and NE1169-1 is serotype STEC O157:H7. The prophages are integrated between *zinT* and *mtfA* with genes of interest highlighted.

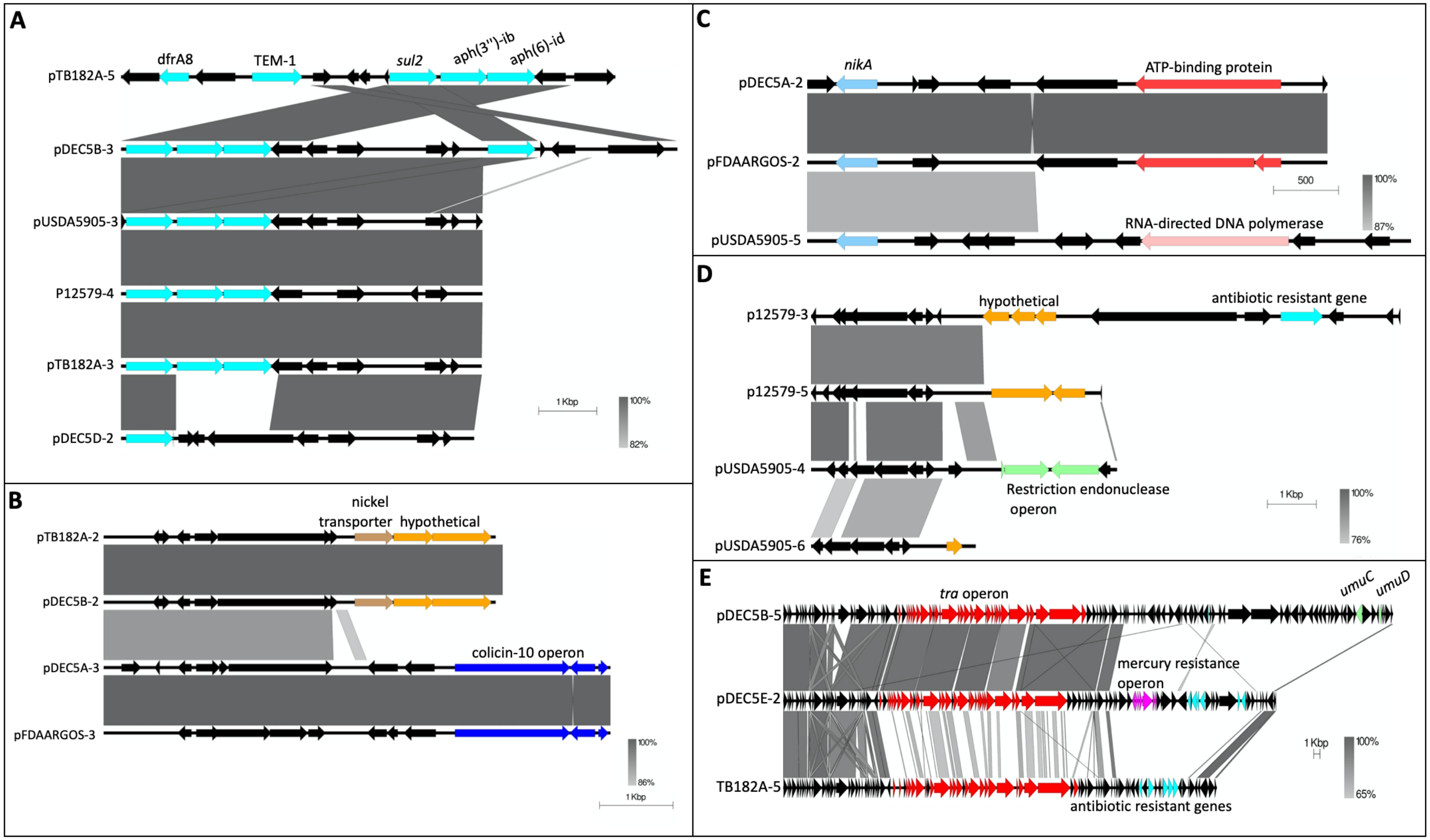

Supplemental Figure S4. Additional plasmid identified in O55:H7 strains. Genes of interest are colored and names are located either above or below its corresponding gene. A) Comparison of the 4 kb to 9 kb plasmid grouping and the antibiotic resistance operon from plasmid TB182A-5 which is a 70 kb plasmid (Panel E). Plasmid pUSDA5905-5 is not closed. B) Comparison of the 5 kb to 6 kb plasmid grouping. C) Comparison of the 4 kb plasmid grouping. D) Comparison of the 3kb to 6 kb plasmid grouping. E) Comparison of the 70 kb to 99 kb plasmid grouping.

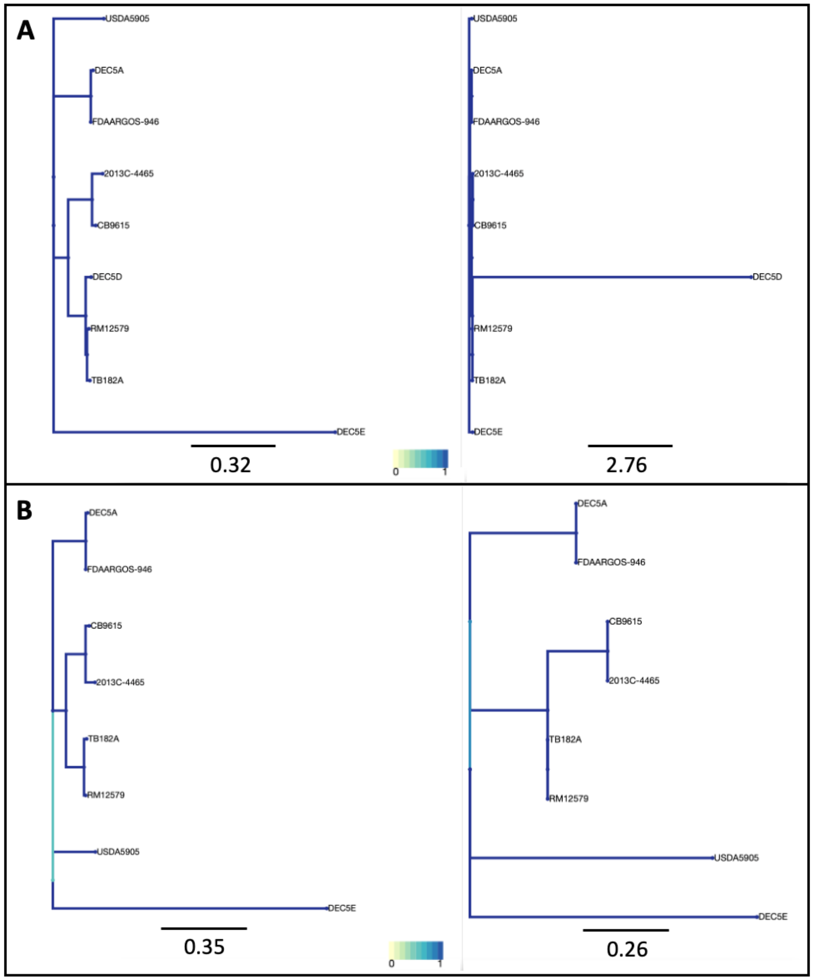

Supplemental Figure S5. Comparison of the phylogenetic trees from the chromosome and pO55 plasmids from O55:H7. A) The comparison of the nine O55:H7 strain that contained a version of the pO55 plasmid. Because the DEC5D plasmid was truncated and had more SNPs than the other eight strains, the branch length is much longer and collapses the tree which makes it harder to see the internal branch node of the other strains. B) The phylogenetic trees minus the DEC5D strain to better show the branching of pO55. The yellow to blue color scheme indicates the similarity of best matching subtrees between the two trees. A score of 1 denotes the subtree structure of the node is identical to the subtree structure of its best corresponding node.

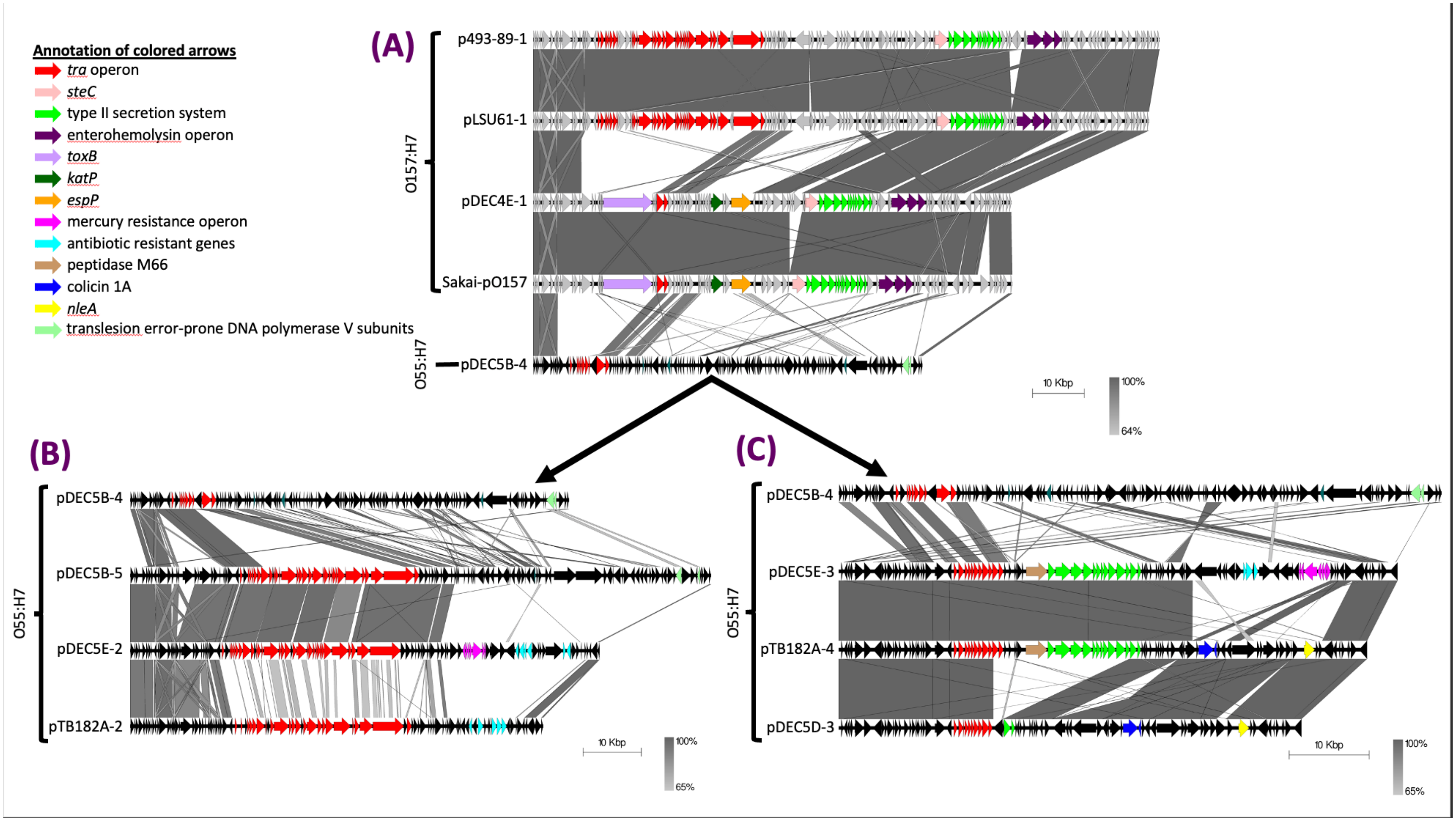

Supplemental Figure S6. Comparison of pDEC5B-4 to other plasmids. (A) comparison of pDEC5B-4 to pO157 plasmids from STEC O157:H7 strains, (B) comparison of pDEC5B-4 to 70 kb to 99 kb plasmids from O55:H7 strains, and (C) comparison of pDEC5B-4 to pO55 plasmids from O55:H7 strains.
